# Epstein–Barr virus serology and gastric cancer incidence and survival

**DOI:** 10.1038/sj.bjc.6604063

**Published:** 2007-11-06

**Authors:** J Koshiol, Y-L Qiao, S D Mark, S M Dawsey, C C Abnet, F Kamangar, E T Lennette, Z-W Dong, P R Taylor

**Affiliations:** 1 Cancer Prevention Fellowship, Division of Cancer Prevention, National Cancer Institute, 6120 Executive Blvd., MSC 7236, Bethesda, MD, USA; 2 Division of Cancer Epidemiology and Genetics, National Cancer Institute, Bethesda, MD, USA; 3 Cancer Institute, Chinese Academy of Medical Sciences, Beijing, People's Republic of China; 4 Department of Preventive Medicine and Biometrics, University of Colorado Medical Center, Boulder, CO, USA; 5 Nectandra Institute, San Ramon, Costa Rica

**Keywords:** EBV, gastric cancer, incidence, survival

## Abstract

Among 185 cases of gastric cancer and 200 controls in Linxian, China, Epstein–Barr virus (EBV) seropositivity was not associated with increased risk of gastric cancer. High EBV nuclear antigen titres were associated with longer survival in cardia cancer cases, possibly due to chance.

Epstein–Barr virus (EBV), an established cause of lymphoma and nasopharyngeal carcinoma, has been detected in approximately 10% of gastric tumours (Young and Rickinson, 2004), leading to speculation that it may also cause gastric cancer.

Previous studies relating EBV to gastric cancer have primarily been tumour-based. The only previous study of prediagnostic EBV serology and gastric cancer incidence (Levine *et al*, 1995) found generally elevated odds ratios (ORs) for EBV seropositivity and gastric cancer, but included only 46 cases. No previous study has evaluated prediagnostic EBV serology and gastric cancer survival.

We prospectively evaluated the associations between baseline EBV serology in relation to (1) subsequent gastric cancer and (2) survival among gastric cancer patients in the Linxian General Population Nutrition Intervention Trial (NIT) cohort in China.

## MATERIALS AND METHODS

The NIT was a cancer chemoprevention trial in Linxian, China, conducted from March 1986 through May 1991, that enrolled 29 584 participants (Blot *et al*, 1993). Follow-up for additional survival is available through May 2001 (Tran *et al*, 2005). We analysed the relation of baseline EBV seropositivity to gastric cancer incidence in a nested case–control study of cardia cancer cases (*N*=102, a random sample of the 435 cases that arose during the trial period), non-cardia cancer cases (*N*=83), and randomly selected controls (*N*=200). Case and control selection methods have been described elsewhere (Mark *et al*, 2000). We also analysed the association between EBV seropositivity and survival among gastric cancer cases.

We tested for IgA antibodies against the EBV viral capsid antigen (VCA IgA) and the diffuse early antigen (EA-D IgA), and for IgG antibodies against the EBV viral capsid antigen (VCA IgG), diffuse early antigen (EA-D IgG), restricted early antigen (EA-R IgG), and EBV nuclear antigen complex (EBNA) using immunofluorescence assays, as previously described (Levine *et al*, 1995). EA-D IgA was not detectable. EBV VCA IgA, EA-D IgG, and EA-R IgG antibodies were detected in <15% of cases and controls and so were categorised as positive or negative. EBV VCA IgG and EBNA antibodies, which were detectable in all subjects, were classified as high if above the median category of antibody titres in controls and low otherwise. VCA IgG antibody titres were also analysed ordinally.

Unconditional logistic regression was used to calculate ORs and 95% confidence intervals (CIs) for prediagnostic EBV serostatus and gastric cancer, controlling for age and gender. Other potential confounders (e.g., smoking, alcohol, *Helicobacter pylori*) were considered but did not affect the estimate for VCA IgG. Kaplan–Meier curves and age- and gender-adjusted Cox models were used to evaluate gastric cancer survival by baseline VCA IgG and EBNA.

## RESULTS

Cases tended to be older than controls and were more likely to be males, smokers, and HP positive (Table 1). Among all subjects, EBV VCA IgA was detectable in 2%, EA-D IgG in 14%, and EA-R IgG in 9%. VCA IgG EBNA and antibody titre levels were above the median in the controls in 37% of all subjects.

Overall, prediagnostic EBV seropositivity was not positively associated with gastric cancer in this population (Table 2). In fact, the direction and magnitude of the associations suggested that EBV seropositivity was associated with decreased risk, especially tumours originating in the gastric cardia. A restricted analysis of cases diagnosed ⩾2 years after serology strengthened the association for high VCA IgG but not EBNA.

**Figure 1:**
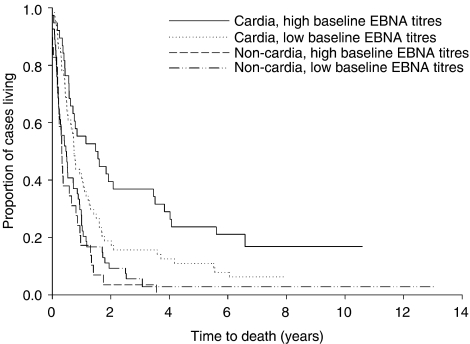
Kaplan–Meier curves for the proportion of gastric cancer cases remaining alive by baseline EBV EBNA serostatus and tumour location.

There was no difference in survival by VCA IgG. Survival was longer among individuals with high *vs* low EBNA (hazard ratio (HR): 0.71, 95% CI: 0.51–0.98). This difference was found in cardia (HR: 0.46, 95% CI: 0.29–0.74) but not in non-cardia cancer cases (HR: 1.18, HR: 0.74–1.88) (Figure 1). Using the likelihood ratio test for multiplicative interaction, the HR for baseline EBNA seropositivity and gastric cancer varied by tumour location (*P*-value=0.03). It is relevant to note, however, that the data become sparse in the tails of the survival curves, so these findings may be due to chance.

## DISCUSSION

We found that prediagnostic EBV seropositivity was not positively associated with gastric cancer in this population and rather may be associated with decreased risk, especially for gastric cardia cancer which, unusually, is more common than non-cardia gastric cancer in Linxian (Blot *et al*, 1993). High baseline EBNA titres were also associated with decreased risk of death after diagnosis among gastric cardia cancer cases. Since EBV seropositivity is established by both viral activity and immune response, these associations may reflect an increased immune response due to viral infection leading to reduced gastric cancer incidence and longer survival.

In contrast to our study, the only previous prospective study reported generally elevated ORs, suggesting an increased risk of gastric cancer with EBV seropositivity (Levine *et al*, 1995). The opposite patterns of risk found in these two studies may be due to (1) chance, with no true underlying association, (2) differences in study populations, or (3) different distributions of tumour location since the previous study included few gastric cardia cancers, while over half the cases in our study had cardia cancer.

Although previous studies of EBV DNA in gastric tumour tissue and survival have produced inconsistent results (Chang *et al*, 2001; Lee *et al*, 2004), some have found improved survival associated with EBV, at least in subgroups (Koriyama *et al*, 2002; Lee *et al*, 2004; van Beek *et al*, 2004). Our association between high EBNA titres and improved survival was also limited to a subgroup, cardia cancers, although this finding may be due to chance. Further, we did not have tumour tissue available to directly test for EBV DNA.

In conclusion, we did not find a positive association between prediagnostic EBV seropositivity and gastric cancer. In fact, there was some evidence that EBV seropositivity was associated with a reduced risk of cancer and a reduced risk of death after diagnosis of cardia cancer. These results support the hypothesis that cardia and non-cardia cancers may be aetiologically different and should be evaluated separately in future studies.

## Figures and Tables

**Table 1:** Selected characteristics and univariate associations with gastric cancer for nested case–control subjects from the General Population Nutrition Intervention Trial cohort

**Characteristic**	***N* Cases (%)**	***N* Controls (%)**
*Age (years)*		
<50	32 (17.3)	58 (29.0)
50–60	79 (42.7)	68 (34.0)
>60	74 (40.0)	74 (37.0)
		
*Gender*		
Male	113 (61.1)	98 (49.0)
Female	72 (38.9)	102 (51.0)
		
*Smoking (regularly for ⩾6 months)*		
No	102 (55.1)	139 (69.5)
Yes	83 (44.9)	61 (30.5)
		
*Alcohol (in last 12 months)*		
Never/rare	143 (77.3)	160 (80.0)
Sometimes/more often	42 (22.7)	40 (20.0)
		
*Helicobacter pylori* a		
Negative	48 (27.8)	73 (45.3)
Positive	125 (72.3)	88 (54.7)

a Numbers do not add up to totals because of missing values.

**Table 2:** Association between prediagnostic EBV seropositivity and development of gastric cancer in the General Population Nutrition Intervention Trial cohort for all cases combined and stratified by tumour location

	**All cases (*n*=185)**		**Cardia (*n*=102)**		**Non-cardia (*n*=83)**	
**EBV variable**	***N* positive cases**	**OR (95% CI)^a^**	***N* positive cases**	**OR (95% CI)^a^**	***N* positive cases**	**OR (95% CI)^a^**
VCA IgA positive	3	0.69 (0.15–3.21)	1	0.46 (0.05–4.20)	2	0.90 (0.15–5.40)
EA-D IgG positive	26	0.95 (0.53–1.70)	13	0.82 (0.41–1.67)	13	1.23 (0.58–2.60)
EA-R IgG positive	11	0.52 (0.24–1.11)	6	0.49 (0.19–1.23)	5	0.55 (0.19–1.57)
High VCA IgG^b^	60	0.72 (0.47–1.09)	32	0.63 (0.38–1.05)	28	0.83 (0.47–1.46)
Restricted^c^	32	0.60 (0.36–0.99)	19	0.54 (0.30–0.99)	13	0.70 (0.33–1.46)
High EBNA IgG	67	0.91 (0.60–1.39)	38	0.97 (0.59–1.60)	29	0.79 (0.45–1.39)
Restricted^c^	42	1.01 (0.62–1.64)	26	1.03 (0.59–1.83)	16	0.90 (0.44–1.84)

a Adjusted for age and gender.

b Odds ratios (ORs) for ordinal VCA IgG: all cases, 0.89 (0.78–1.02); cardia cases, 0.86 (0.74–1.00); non-cardia cases, 0.96 (0.80–1.14).

c Restricted to cases diagnosed after 2 years of follow-up.
